# Label-Free Uric Acid Estimation of Spot Urine Using Portable Device Based on UV Spectrophotometry

**DOI:** 10.3390/s22083009

**Published:** 2022-04-14

**Authors:** Tsung-Jui Lin, Kai-Ting Yen, Chien-Fan Chen, Shuo-Ting Yan, Kuan-Wei Su, Ya-Ling Chiang

**Affiliations:** 1 Taiwan RedEye Biomedical Inc., Hsinchu City 300, Taiwan; ray.lin@redeyebmi.com (T.-J.L.); styan@redeyebmi.com (S.-T.Y.); 2 Department of Electrophysics, National Yang Ming Chiao Tung University, Hsinchu City 300093, Taiwan; df900035.sc06@nycu.edu.tw (K.-T.Y.); yixin.sc09@nycu.edu.tw (C.-F.C.); sukuanwei@nycu.edu.tw (K.-W.S.); 3 College of Artificial Intelligence, National Yang Ming Chiao Tung University, Tainan City 711010, Taiwan

**Keywords:** uric acid, UV spectrophotometry, spot urine

## Abstract

The maintenance of uric acid levels is crucial for the human body. In this study, the feasibility of using portable ultraviolet (UV) spectrophotometry to measure the uric acid of spot urine without the need to add reagents has been demonstrated for the first time. UV spectral analysis has been used to inspect the uric acid concentration in urine. It is found that the absorption spectrum of urine has a high correlation with the concentration of uric acid at a wavelength of around 290–300 nm. Uric acid levels measured with a spectral analyzer compared to uric acid concentrations measured with a traditional biochemical analysis showed good agreement. The portable prototype is label-free and capable of displaying the inspection result of each measurement within 10 s. In the long run, this device can assist people in checking uric acid levels of spot urine with higher frequency and can adjust diet or medication in real time for more efficient health management.

## 1. Introduction

The major sources of uric acid in the human body are decomposed body tissues and the food consumed. After liver metabolism, uric acid exists in blood or is excreted through urine. If the concentration of uric acid in human urine or blood is too high, uric acid may be crystalized and deposited on the joints and/or kidneys. Joints rub against the uric acid crystals, inducing joint redness, swelling, and heat, commonly known as gout. If the uric acid level is not under control, severe symptoms such as joint deformation could appear. When uric acid is deposited on the kidneys, kidney stones form. If the concentration of uric acid in the urine is too low, it may be an early warning of impaired kidney function.

Gout is one of the most common inflammatory arthritides globally. The prevalence of gout all over the world can range from <1% to 6.8%, depending on the population and the method employed in their studies [1]. The disease has been reported to be associated with abnormal levels of uric acid in the human body. Excessive uric acid can cause the formation of uric acid crystals. It deposits in tendons, joints, and other tissues [2]. The accumulation of uric acid crystals in joints causes patients severe pain. Other diseases and complications related to gout include chronic renal disease [3] and heart vascular disease [4]. Therefore, it is critical to monitor uric acid levels continuously for gout patients and people with high risk of gout. In addition, abnormal serum uric acid levels could also be a risk factor for fatty liver [5] and Parkinson’s disease [6], and abnormal urinary uric acid levels could be a risk factor for nephrolithiasis [7] and dehydration [8], as well as an indication of high-protein and high-purine diets [9,10].

The characterization of 24-h urinary uric acid excretion and serum uric acid is critical to the diagnosis of gout. The normal range of 24-h urinary uric acid is 250–750 mg [11]. However, the collection of 24-h urine is inconvenient for both patients and medical workers. For example, a 24-h urinary uric acid test requires storing urine samples at 4–6 °C [11]. During the 24-h urine storage process, the supersaturation of the stored samples may lead to the underestimation of urinary uric acid concentration [12]. A 24-h period is not short for a patient. Missing collection of urine samples often occurs. The complexity of storage, contamination prevention, and carrying the collection container could confuse the patients, which further decreases their will to perform the test. Improper specimen collection of urine often results in incorrect results of 24-h urine tests [13]. Serum uric acid test is invasive. The pain of the tests may induce psychological pressure on patients who need daily uric acid level monitoring. Additionally, the uric acid level in the serum and the uric acid level excreted from urine can be different at the same period of time [14]. From a preventive medicine point of view, it is worthwhile to develop a convenient and comfortable method for gout patients and people who are used to consuming high purine diets [9]. Continuous and frequent uric acid detection is critical to maintain a healthy uric acid level.

Spot urine is a sample with high accessibility and usability. Some studies have suggested using spot urine to estimate uric acid excretion [15,16], selecting gout patients for further medication [17,18], and guiding the therapy of hyponatremia [19].

In clinical practice, the primary method used to measure the concentration of uric acid is the enzymatic method. Uricase is first used to oxidize uric acid into allantoin and hydrogen peroxide, and then peroxidase is used to produce quinoneamine dye. The main absorption wavelength of the quinoneamine dye, as a quantified target, used to determine uric acid level, will be around 520 nm [20,21]. However, recent studies on the ultraviolet (UV) spectrum of uric acid showed that it has its own significant absorption characteristic [22,23], which indicates the possibility of developing a label-free concentration measurement method of uric acid in urine.

This study reports a specially designed portable device to measure the concentration of uric acid in spot urine based on UV spectrophotometry. The performance of the portable device was tested by comparing it with a desktop UV spectral analyzer system and a standard biochemical analyzer. The device developed in this study mainly detected uric acid at 290 nm wavelength and did not need to add chemical solvents or labels. In the future, it will be possible to realize real-time uric acid monitoring of spot urine using a compact and portable UV spectrophotometer, enabled with a telemedicine system. Real-time uric acid monitoring will help users with alarms to adjust diet and medication in real time, which will further reduce the incidence of gout and help maintain quality of life.

## 2. Materials and Methods

**Figure 1:**
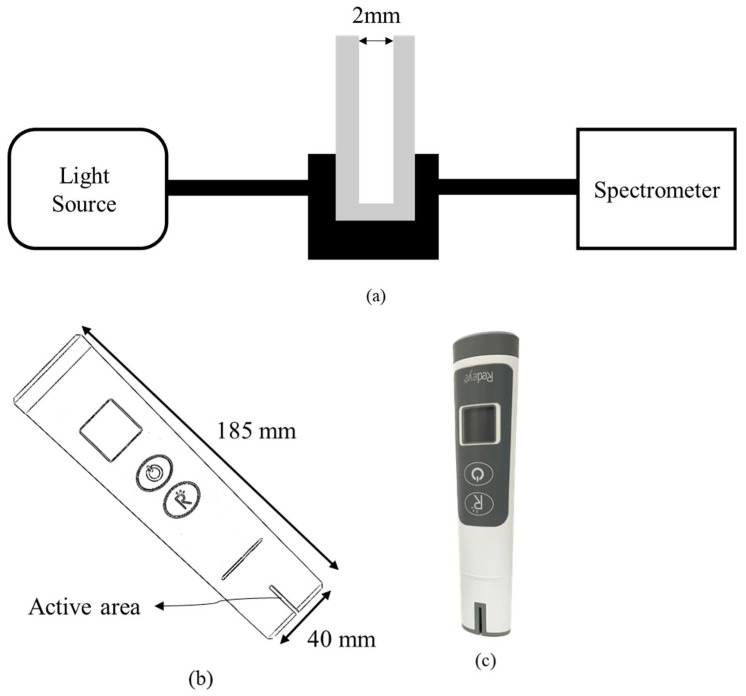

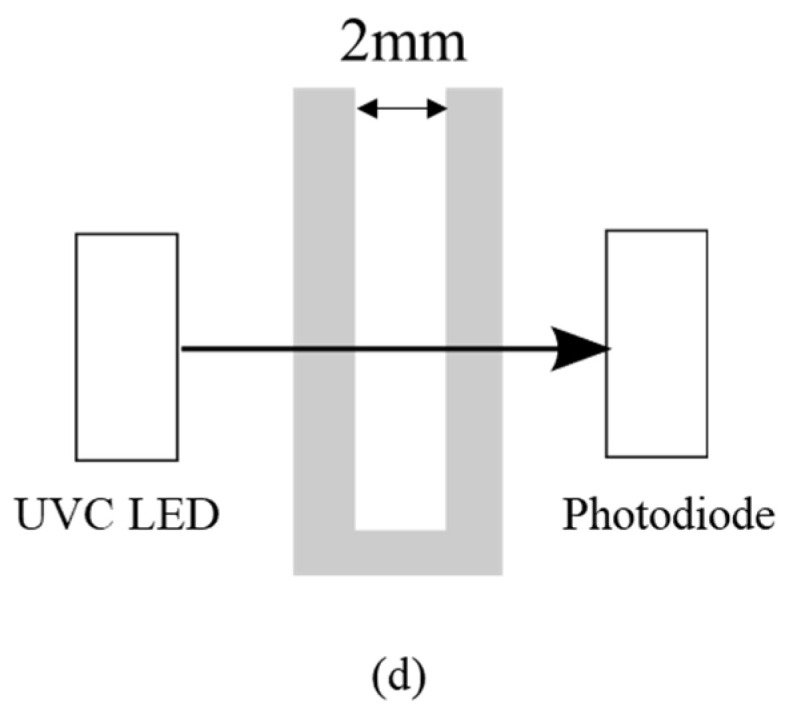
(**a**) The spectral analyzer system set up; (**b**) the portable proof-of-concept prototype for testing uric acid in urine; (**c**) the photograph of the real device; (**d**) the structure of the active area.

First, urine samples from 10 volunteers were tested using the commercialized biochemical analyzer and the UV spectral analyzer. A urine sample of 3 mL was collected and placed into the biochemical analyzer for analysis. The biochemical analyzer used in the study was SIEMENS ADVIA Chemistry XPT (Siemens Healthcare Diagnostics, Tarrytown, NY, USA). Uricase and uric acid reacted and converted to allantoin and hydrogen peroxide. The hydrogen peroxide reacted with peroxidase to create a red dye. The concentration of uric acid can be determined by the absorbance of the red solution at 546 nm [24]. At the same time, 6 mL, 4 mL, and 3 mL urine samples were collected and added into 54 mL, 56 mL, and 57 mL of pure water for the UV spectral analyzer. Additionally, 98 mL of pure water was added to 100 mg of uric acid powder (Sigma-Aldrich, St. Louis, MO, USA) and 2 mL 2 M NaOH solution to form a solution concentration of 100 mg/dL of uric acid. In addition, 1 mL, 2 mL, 3 mL, 4 mL, 5 mL, 6 mL, 7 mL, and 8 mL of uric acid solution of 100 mg/dL were collected and added into 99 mL, 98 mL, 97 mL, 96 mL, 95 mL, 94 mL, 93 mL, and 92 mL pure water to form a set of uric acid calibration solutions of 1 mg/dL, 2 mg/dL, 3 mg/dL, 4 mg/dL, 5 mg/dL, 6 mg/dL, 7 mg/dL, and 8 mg/dL for the UV spectral analyzer, respectively. The system configuration of the UV spectral analyzer used in this study is shown in Figure 1a. The spectrometer was GIE Optics Mars Hs2000+ with a resolution of <1.5 nm, and the light source was both halogen and deuterium lamps. The aqueous sample was placed into a cuvette with a 2 mm optical path, and the cuvette was placed into a cuvette holder with two collimators for connecting optical fibers. Two optical fibers were used to transmit and receive the light signal from the light source to the cuvette and the spectrometer. The light intensity was attenuated when passing through an object. Absorbance can be defined as the following formula:

(1) $$A=-\log\left(\frac{I}{I_{0}}\right)$$

*A* is the absorbance, *I*_0_ is the incident light intensity, and *I* is the transmitted light intensity. In addition, Beer–Lambert’s law defines the relationship between absorbance and concentration by the following formula:

(2) A=εcL

The absorbance has a linear relationship with the concentration (*c*), the molar attenuation coefficient (ε), and the optical path (*L*).

In this study, a proof-of-concept prototype according to the above spectral analyzer structure to verify the feasibility of portable devices for detecting uric acid in urine has been demonstrated for the first time. Figure 1b illustrates the portable proof-of-concept prototype for testing uric acid in urine. A photograph of the prototype used in this study is shown in Figure 1c. The active area contained light sources and optical sensors with an optical path of 2 mm for liquid solution, as shown in Figure 1d. The light source used was a UVC LED of 290 nm with the full width at half maximum (FWHM) of 14 nm. The optical sensor used was a photodiode with high UV responsivity. Using the portable setup with UV spectral detectability, the active area was defined to immerse into the aqueous solution to inspect.

## 3. Results and Discussion

**Figure 2:**
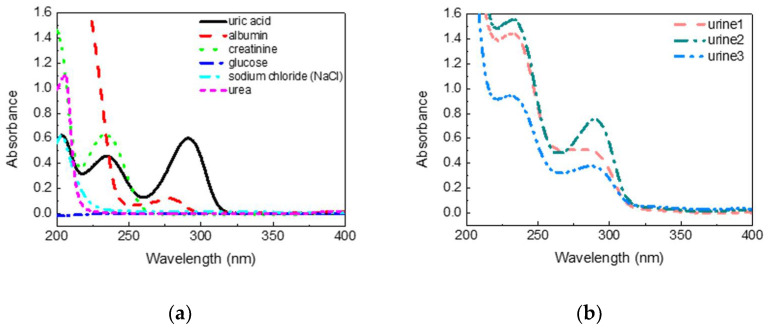
(**a**) The absorption spectra of common substances in the urine; (**b**) the absorption spectra of diluted urine of three different people.

Urine is a mixture of uric acid and other substances [25]. The UV absorption spectra of common substances in urine are shown in Figure 2a. All the samples were measured in a water solution ten times each. The concentrations of substances shown here were 5 mg/dL of uric acid, 100 mg/dL of albumin, 5 mg/dL of creatinine, 200mg/dL of glucose, 200 mg/dL of sodium chloride, and 150 mg/dL of urea. It was found that uric acid and creatinine had strong absorption characteristics even at low concentrations, and only one of the absorption peaks of uric acid was longer than 280 nm in the UV band. Other substances showed apparent absorption for the wavelength shorter than 280 nm, whereas the albumin showed a protein absorption peak around 278 nm. Uric acid had absorption peaks at 205 and 230 nm, which is highly interfered. It is inferred that the absorption spectrum at 290–300 nm had low interference for uric acid detection in urine. Figure 2b shows the absorption spectra of diluted urine of three people, with the largest differences in ten people. Three urine samples were diluted 10 times by pure water due to the high absorbance of urine in the UV band. It was found that urine had a distinct spectral characteristic near 290 nm, which corresponded to the absorption peak of uric acid. Therefore, it may be feasible to inspect the uric acid concentration in urine by absorbance analysis around 290–300 nm. A broader investigation of substances, such as drugs, and clinical research will be carried out to verify if taking medicine or other behaviors will cause interference [26].

**Figure 3:**
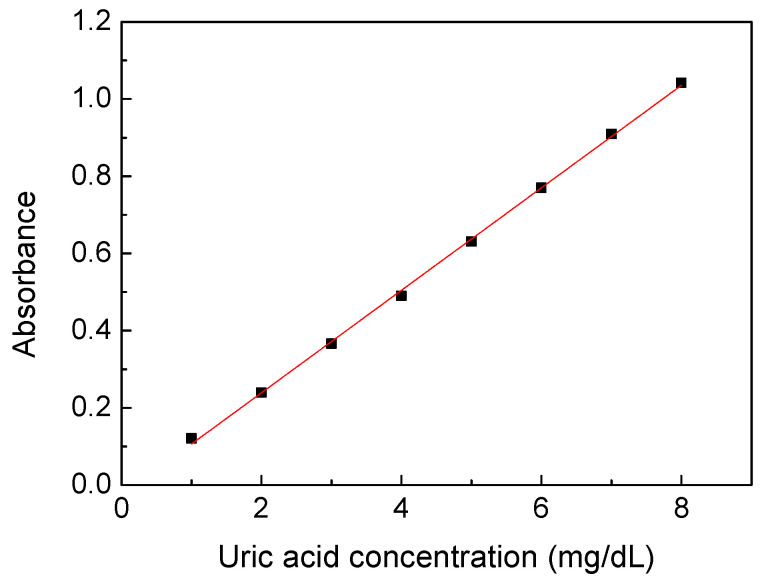
A linear relationship between the absorbance and concentration of the uric acid calibration solution at wavelength 300 nm.

A calibration process is required to correct the relationship between uric acid concentration and absorbance and compensate for the tolerance of the measurement system. Figure 3 shows the relationship between the absorbance of the calibration solution prepared by uric acid powders at wavelength 300 nm and its concentration. The calibration solution was prepared at concentrations of 1–8 mg/dL because the uric acid concentration would be measured at the range when the urine sample was diluted 10 to 20 times in the subsequent experiments. Each sample was measured 10 times, and the standard deviation of the data points was below 0.002. It showed nearly ideal linearity between the absorbance and concentrations of the uric acid calibration solution to acquire the linear equation with a high coefficient of determination of 0.9991 (slope of 0.1327, intercept of −0.0266). It also proved that the desktop spectral analyzer system was stable and had only a small tolerance.

**Figure 4:**
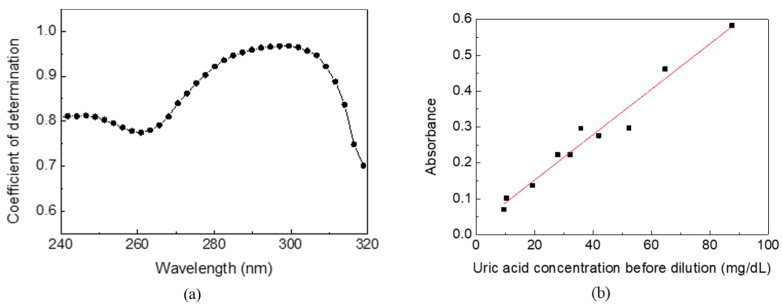
(**a**) The coefficient of determination distribution between the absorbance and the uric acid concentration of urine samples at wavelength 240–320 nm. (**b**) The relationship between the absorbance and the uric acid concentration of urine samples at 300 nm. The coefficient of determination reached its highest level at 0.9633.

The absorption spectra and uric acid concentrations of urine samples from 10 volunteers were analyzed to verify the optimized detecting wavelength and feasibility in the real world. As the control group, the uric acid concentrations of urine samples measured by the biochemical analyzer were distributed between 9–90 mg/dL. Meanwhile, each of the 10 urine samples of 1 mL were added to 19 mL of pure water (i.e., 20-times dilution), followed by a traditional desktop spectral analyzer measurement. The coefficient of determination, R^2^, between the absorbance and the uric acid concentration of urine samples at wavelength 240–320 nm is shown in Figure 4a. The *X*-axis is a continuous wavelength scanned from 240 nm to 320 nm, and the *Y*-axis is a coefficient of determination at each wavelength. A higher coefficient of determination implies higher correlation and specificity for detecting uric acid in human urine. It shows that the optimized detecting wavelength is around 300 nm, consistent with the aforementioned low interference at wavelengths longer than 280 nm. The coefficient of determination decreases rapidly at wavelengths above 305 nm due to the decreasing absorption of uric acid. Figure 4b shows the linearity between the absorbance and the uric acid concentration of urine samples at 300 nm with the highest R^2^ of 0.9633. The standard deviation of the data points is below 0.002. Compared to the biochemical analyzer with high specificity that only uric acid reacts on uricase to catalyze the biological reaction, other substances and impurities in urine caused accumulated absorption and turbidity and a corresponding positive intercept of 0.0257. The concentration and composition ratio of other substances and impurities also reduced the coefficient of determination. However, the experimental results of UV spectrophotometry indicate the feasibility of using a single detecting wavelength to estimate the uric acid concentration in human urine.

**Figure 5:**
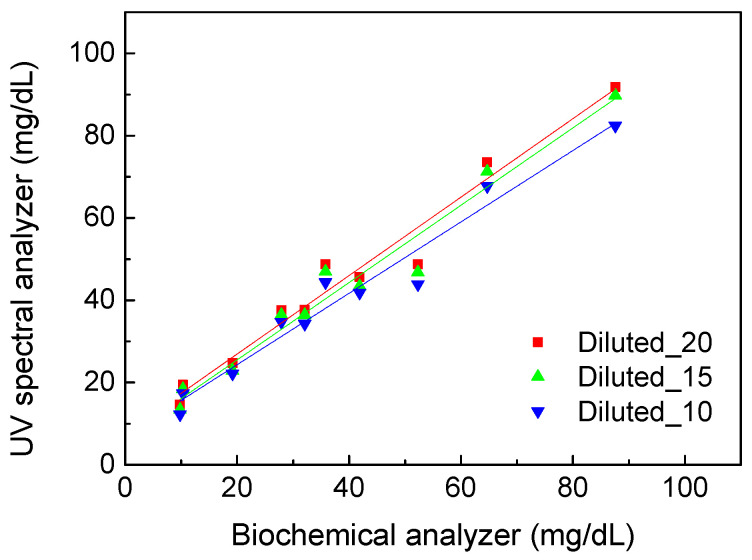
The comparison of uric acid concentration detection results using the desktop spectral analyzer with respect to the biochemical analyzer. The blue (Diluted_10) data represent the R^2^ of 0.9571, whereas green (Diluted_15) and red (Diluted_20) data represent the R^2^ of 0.9605 and 0.9630, respectively.

Further, we measured and calculated uric acid concentrations based on the calibration line shown in Figure 3. Figure 5 shows the comparison results of uric acid concentration detected using the UV spectral analyzer with calibration and the biochemical analyzer. All the urine samples were diluted 10, 15, and 20 times to understand the influence of the dilution ratio. The standard deviation of all data points was below 1.7 mg/dL. The R^2^ of 0.9571 was obtained under 10-fold dilution. The highest R^2^ of 0.9630 was obtained under 20-fold dilution, and a similar R^2^ of 0.9605 was obtained under 15-fold dilution. Therefore, anything beyond 15-fold dilution will not contribute significantly to the accuracy of the UV spectral analysis method. On the other hand, the values of R^2^ were all higher than 0.95 for the three dilution ratios. That means this method can be used to quantify uric acid in urine. The spectral analysis method has the feasibility to be developed for real-time monitoring of uric acid with IoT technology connected to mobile devices.

**Figure 6:**
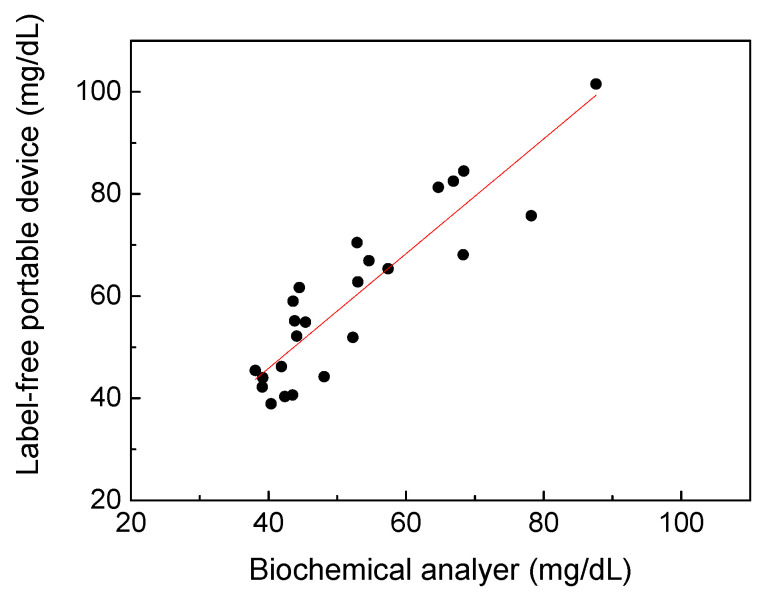
The uric acid concentration detection results using the label-free portable device with respect to the biochemical analyzer. The coefficient of determination is 0.8083.

According to the UV spectral analysis results, a portable prototype shown in Figure 1b has been developed for uric acid estimation of spot urine without adding labels in this study. Here, a total of 58 urine samples were tested to compare the accuracy of the portable prototype with that of the biochemical analyzer. Due to prototype limitations and the consideration of convenience in daily life, the urine samples were diluted six times and could not be measured at uric acid concentrations below 40 mg/dL. Therefore, only 24 cases are shown in Figure 6. The standard deviation of the data point ranges was found to be 0.75 mg/dL. The coefficient of determination between the label-free portable prototype and the biochemical analyzer was 0.8083, indicating the feasibility of spectral analysis performed by a portable device based on UV spectrophotometry to detect uric acid in urine without enzymes. The wavelength of the selected LED was 290 nm instead of 300 nm due to the limitation of LED availability. Following works will be focused on finding cost-effective, compact, and available light sources closer to 300 nm, and conducting further clinical research on the variations of the absorption spectrum of urine to lead the results of this device to be closer to the biochemical analysis. The portable device is not intended to replace biochemical analyzers, but to create the opportunity and possibility to meet the need of point-of-care and real-time monitoring of uric acid in urine in clinical and general-wellness applications.

## 4. Conclusions

A high coefficient of determination between the uric acid concentration and the UV absorption of urine has been shown in this study. The uric acid level in urine can be detected by a non-invasive spectral analysis that does not need any chemical labeling. With the calibration prepared by uric acid powder solution, the accuracy and correlation of spectral analysis can be enhanced. Additionally, a portable device developed for label-free uric acid estimation of spot urine has been demonstrated for the first time. It shows a high correlation compared with traditional biochemical analysis. As a result, it is feasible to optimize for higher accuracy and integration via Internet of Things (IoT) technology for telemedicine application, as well as adjusting diet or medication in real-time to lower the risk of gout and maintain a healthy level of uric acid in urine and blood.

## Data Availability

The data presented in this study are available on request from the corresponding author, providing the access does not interfere with the conditions provided by the ethics committee.
